# Length heterogeneity at conserved sequence block 2 in human mitochondrial DNA acts as a rheostat for RNA polymerase POLRMT activity

**DOI:** 10.1093/nar/gkw648

**Published:** 2016-07-19

**Authors:** Benedict G. Tan, Frederick C. Wellesley, Nigel J. Savery, Mark D. Szczelkun

**Affiliations:** DNA-Protein Interactions Unit, School of Biochemistry, University of Bristol, Bristol BS8 1TD, UK

## Abstract

The guanine (G)-tract of conserved sequence block 2 (CSB 2) in human mitochondrial DNA can result in transcription termination due to formation of a hybrid G-quadruplex between the nascent RNA and the nontemplate DNA strand. This structure can then influence genome replication, stability and localization. Here we surveyed the frequency of variation in sequence identity and length at CSB 2 amongst human mitochondrial genomes and used *in vitro* transcription to assess the effects of this length heterogeneity on the activity of the mitochondrial RNA polymerase, POLRMT. In general, increased G-tract length correlated with increased termination levels. However, variation in the population favoured CSB 2 sequences which produced efficient termination while particularly weak or strong signals were avoided. For all variants examined, the 3′ end of the transcripts mapped to the same downstream sequences and were prevented from terminating by addition of the transcription factor TEFM. We propose that CSB 2 length heterogeneity allows variation in the efficiency of transcription termination without affecting the position of the products or the capacity for regulation by TEFM.

## INTRODUCTION

Human mitochondria contain multiple copies of a circular, double-strand mitochondrial DNA (mtDNA) genome that encodes core proteins of the electron transport chain, as well as tRNAs and rRNAs necessary for organelle-specific translation. A distinctive feature of mtDNA replication is that due to the absence of a dedicated mitochondrial primase, RNA primers for initiation of replication by DNA polymerase γ appear to be provided by the RNA polymerase, POLRMT (1–4). It has been suggested that these primers arise from a switch in POLRMT activity that causes RNA transcripts to remain bound to the template as discontinuous but persistent RNA:DNA hybrids, termed ‘R-loops’. At some locations, R-loops may be formed following transcription of homopolymeric guanine (G)-tracts (5–8). Guanine-stabilized R-loops appear to be widespread, also being found in the nucleus, and play roles in DNA methylation, histone modification, transcription, replication initiation and immunoglobulin class-switch recombination (9–11). Here, we investigated how naturally-occurring variations in the length of a specific G-tract can affect transcription by human POLRMT.

**Figure 1.**
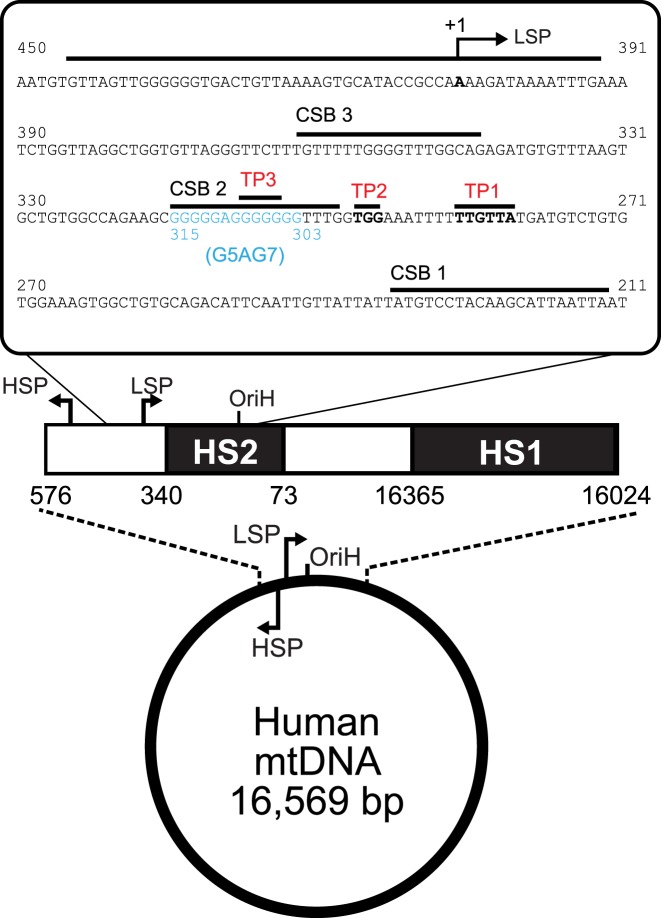
The DNA sequence of the human mitochondrial NCR encompassing the light strand promoter and Conserved Sequence Blocks 1, 2 and 3. The control region of human mtDNA is indicated, with the locations of LSP, HSP, O_H_, and HS1 and HS2 highlighted. The nucleotide from 211 to 450 of the revised Cambridge Reference Sequence (rCRS) (47), are shown, with the locations of the LSP and of CSB 1, 2 and 3 highlighted (47). The locations of the 3′ ends of the transcription products observed here are shown (TP1, 2 and 3). We analysed length heterogeneity due to changes to the G-tract between nucleotides 303–315, with the remainder of the sequence remaining the same.

The mechanism of human mtDNA replication is not fully understood and a number of conflicting models have been proposed (12–16). In many of the models, initiation of heavy strand synthesis occurs at an origin (O_H_) found within an ∼1.1 kb intergenic non-coding region (NCR) (Figure 1). (The two strands of mtDNA are termed heavy and light due to different buoyant densities under centrifugal gradients). The NCR also contains promoters for transcription of the light strand or heavy strand (LSP or HSP, respectively). Full length transcripts produced from the LSP or HSP are polycistronic and are processed following transcription. However, LSP transcription events frequently terminate prematurely within the NCR at a series of Conserved Sequence Blocks (CSB 3, CSB 2 and CSB 1; Figure 1). For example, transcription of the adenine-interrupted, discontinuous G-tract of CSB 2 leads to formation of a hybrid quadruplex between the RNA transcript and non-template DNA that causes ‘premature termination’ of POLRMT (5–8). This may then result in formation of R-loop structures that provide free 3′ ends to prime subsequent DNA synthesis (1,4,17–21). Preventing quadruplex formation reduces both POLRMT premature termination and R-loop formation (5,20). An intriguing recent observation is that the transcription factor TEFM inhibits transcription termination at CSB 2 by increasing the stability of the POLRMT elongation complex, and thus may act to regulate R-loop formation and handover between transcription and replication machinery (22,23).

mtDNA accumulates mutations much faster than nuclear DNA, leading to regions of hypervariable sequence within the NCR (HS1 and HS2, Figure 1) and homopolymeric G-tracts are particular hot spots for sequence variation (24–26). Many studies have noted that CSB 2 (which is part of HS2) can vary in the number of residues in the first or second G-run of the discontinuous G-tract (25,27–33). Such length heterogeneity can occur between individuals, between tissues of an individual, or even within single cells, and has been used to designate human haplogroups for studying evolution, genealogy, anthropology, and forensics. Where variation occurs within one cell, tissue or organism (but not between individuals), this is sometimes alternatively referred to as ‘length heteroplasmy’. High levels of length heterogeneity have been observed in hair follicle cells and in oocytes, suggesting a role in rapidly dividing cells (34–38). Differences have also been linked to some cancers (39,40). Continuous G-tracts at CSB 2 are also observed, where the central adenine residue has presumably been mutated (28,41–44). However, there is sometimes ambiguity in the definition of CSB 2 mutations [highlighted by (45)] and it is unclear whether there is any global correlation between variations in length of the first and second G-run. A better understanding of the extent of CSB length heterogeneity in the human population is important in interpreting the range of possible effects on POLRMT transcription.

Most of the *in vitro* transcription studies with purified proteins have used mitochondrial DNA substrates based on the revised Cambridge Reference Sequence (rCRS), where the discontinuous CSB 2 sequence is five guanines followed by seven guanines (G5AG7) (46,47) (Figure 1). However, haplogroup studies observe that other variants are more common, in particular those with six guanines in the first run and seven to nine guanines in the second run. How might such length heterogeneity affect the activity of POLRMT? In general terms, increasing the number of guanine residues would be expected to increase quadruplex stability. In agreement with this, (23) showed that a G6AG8 sequence increased transcription termination relative to a shorter G5AG7 sequence. It has also been shown using the bacteriophage T7 RNA polymerase, which is structurally-related to POLRMT, that length increases in the second G-run of discontinuous sequences (G6AG5 to G6AG15) or in continuous sequences (G9 to G13) produces a general trend of increased premature termination (43). However, it is still not clear how the full range of human CSB 2 length heterogeneity would affect POLRMT in terms of the magnitude and location of transcription termination.

To investigate the extent of length heterogeneity at CSB 2 sequences in human mtDNA, we surveyed sequences deposited in GenBank. We then used *in vitro* assays with purified recombinant human POLRMT to measure how the distribution of observed length heterogeneity affected the levels and location of transcription termination. We show that while length heterogeneity of both discontinous and continuous G-tracts can alter the amount of transcription termination at CSB 2, it does not radically alter the 3′ positions of the resulting terminated transcripts. We also show that the anti-termination effect of TEFM seen previously (22,23) is insensitive to changes in sequence length or identity at CSB 2. We therefore propose that length heterogeneity can modulate the amount of transcription termination at CSB 2 in a controllable manner without drastically affecting the positioning of the termination products, in turn allowing regulation of downstream processes such as replication initiation.

## MATERIALS AND METHODS

### Genome database analysis

Complete or partial human mitochondrial DNA sequences were downloaded from GenBank by searching for either complete human mitochondrial genomes (28 726 sequences) or partial human mitochondrial genome sequences that included the terms ‘control region’ or ‘hypervariable segment’ under ‘[All fields]’ (63 530 sequences). We could not directly filter our search to include either ‘CSB’ or ‘Conserved Sequence Block’ as these terms are generally not present in GenBank definitions or features. Therefore many of the downloaded partial sequences did not include the region covering CSB 2—for example, they are sequences of HS1 alone. The downloaded sequences were analysed without further curation using prfectBLAST (48), a standalone version of BLAST (49). Sequences of bases corresponding to the region 294–325 of the rCRS were used as search strings (based on the minimum of 25 residues required for a search). Each string had varying numbers of guanines in the first and/or second G-runs (between 1 and 12). Algorithmic parameters were set to ensure hits with 100% sequence identity: Maximum target sequence, 20 000; expected threshold, 500; word size, 7; scoring, default; all filters, off; nident; perc_identity 100; num_descriptions, 30 000; evalue, 0.001. This process was then repeated with the search string modified so that the central adenine at 310 was replaced by either cytosine, thymine or guanine. Sequences that included CSB 2 but which varied from the search string outside of the CSB2 G-tract (e.g. due to single nucleotide mutations) would be overlooked by our search strategy.

### Proteins

Human POLRMT with the mitochondrial signal peptide removed (aa 41–1230, Supplementary Figure S1A) was purified as an N-terminal His_6_ construct based on (50). Human TFAM with the mitochondrial signal peptide removed (aa 43–246, Supplementary Figure S1B) was also purified as an N-terminal His_6_ construct using a similar protocol. Full-length human POLRMT cDNA cloned into pBlueScriptR was obtained from Geneservice Ltd (ID:5264127). The region encoding residues 41–1230 was transferred into pET-DuetI (Novagen) using SacI and KpnI sites. Full length human TFAM cDNA cloned into pCR4-topo was obtained from Geneservice Ltd (ID:8992082). The region encoding residues 43–246 was amplified using PCR primer pair 5′-GGAATTCCATATGTCATCTGTCTTGGCAGG-3′ and 5′-CCGCTCGAGTTAACACTCCTCAGCACC-3′ that placed an NdeI site upstream of the coding region and an XhoI site downstream. The resulting PCR product was inserted between the NdeI and XhoI sites of pET28a (Novagen). To express POLRMT or TFAM, *Escherichia coli* BL21(DE3) cells were transformed with the appropriate expression plasmid. Cultures were grown in LB at 37°C. At an OD_600_ ∼0.5, expression was induced with 0.1 mM isopropyl-β-d-thiogalactopyranoside (IPTG) for POLRMT, and 1 mM IPTG for TFAM, and cells harvested after 4 and 1 h respectively. Cells were lysed by sonication in 50 mM sodium phosphate, pH 8.0, 300 mM NaCl and 20 mM imidazole, plus protease inhibitor (Roche complete, EDTA-free). Both proteins were purified by affinity chromatography on a nickel Histrap HP column (GE Healthcare), eluted via a 20–500 mM imidazole gradient, followed by a Hitrap Heparin HP column (GE Healthcare), eluted via a 0.15–1 M NaCl gradient. Proteins were dialysed into storage buffer [POLRMT: 50 mM sodium phosphate, pH 8.0, 50% (v/v) glycerol. TFAM: 10 mM Tris–Cl, pH 8.0, 1 mM DTT, 100 mM NaCl, 20% (v/v) glycerol], concentrated by ultrafiltration, and stored in aliquots at −80°C.

Human TEFM with the mitochondrial signal peptide removed (aa 36–360) was purified as a C-terminal His_6_ construct based on (22) (Supplementary Figure S1C). A gene encoding TEFM 36–360, codon-optimized for *E. coli* protein expression, was supplied by Eurofins (Supplementary Figure S1D), and cloned into the NdeI–XhoI sites of pET24b to make pET24b-TEFM. *E. coli* BLR(DE3) cells were transformed with pET24b-TEFM. Cultures were grown in LB at 37°C. At an OD_600_ ∼0.4, expression was induced with 0.2 mM IPTG and cells were harvested after overnight incubation at 16°C. Cells were lysed by sonication in lysis buffer [100 mM HEPES–HCl, 0.5 M NaCl, 10 mM imidazole, 10% (v/v) glycerol, 1 mM DTT, pH 7.5] plus protease inhibitor (Roche complete, EDTA free) and 9 μg/ml phenylmethane sulfonyl fluoride. His_6_-TEFM was purified by affinity chromatography on a 5 ml Histrap HP column (GE Healthcare), eluted via a 0.04–1 M Imidazole gradient. Following dialysis against lysis buffer without imidazole but with 0.25 M NaCl added, the protein was further purified using a 20 ml HiPrep Heparin 16/10 FF column (GE Healthcare), eluted via a 250–1 M NaCl gradient. Pooled His_6_-TEFM fractions were dialysed against storage buffer (20 mM HEPES–HCl, 10% (v/v) glycerol, 300 mM NaCl, 2 mM DTT, pH 7.5), and subjected to an additional size-exclusion chromatography step on a HiLoad 16/60 Sephadex S200 column. Pure TEFM was concentrated by ultrafiltration and stored in aliquots at −80°C.

Purified human TFB2M (aa 31–396) was supplied by Enzymax LLC. All other enzymes were from New England Biolabs unless stated.

### DNA substrates

All PCR reactions used Phusion DNA Polymerase (Thermo Fisher Scientific). p*lac*CONS-Spe was generated from p*lac*CONS (51) by inserting oligonucleotides containing a SpeI site into the KpnI site (Supplementary Figure S2). The NCR region (nucleotides 683–15 910) was PCR amplified from mtDNA extracted from human RPE1 cells (a gift of Jon Lane), using primers in Supplementary Figure S2, and the product inserted into the SpeI site of p*lac*CONS-Spe. The NCR sequence was converted into that of the rCRS (47), using several rounds of site-directed QuikChange mutagenesis (Agilent) with the primer pairs indicated in Supplementary Figure S2, to make pGC-NCR(rCRS). G-tract length heterogeneity mutations, as well as all other modifications to substrate sequences mentioned in this work, were then introduced using inverse PCR with the primer pairs indicated in Supplementary Figures S3 and S4.

Linear DNA templates for transcription assays comprising nucleotides 509–16478 of the rCRS (102 bp upstream and 500 bp downstream from the LSP) were PCR amplified from the relevant plasmid using 5′-GTAGGATGGGCGGGGGTTGTATTGATGAG-3′ and 5′-GCTAAAGTGAACTGTATCCGACATCTGGTTCCT-3′. Impurities from the PCR reaction were removed using a PCR cleanup kit (Qiagen).

### Transcription assays

Prior to the addition of nucleotides, all reactions were set up on ice. DNA templates were mixed with the appropriate proteins in Reaction Buffer (20 mM Tris–Cl, pH 8.0, 10 mM MgCl_2_, 1 mM DTT, 0.1% (v/v) Tween 20, 0.1 mg/ml bovine serum albumin (BSA)). Following 2 min pre-equilibration at 35°C, transcription was initiated by the addition of an NTP mix. Final reaction conditions were 150 μM GTP, 150 μM CTP, 150 μM ATP, 10 μM UTP, 0.033 μM α-^32^P-UTP (3000 Ci/mmol), 12.5 nM DNA template, 50 nM POLRMT, 50 nM TFAM, 50 nM TFB2M, and, where indicated, 100 nM TEFM monomer. Reactions were stopped after 30 min by the addition of 1/5 volume of stop buffer (0.1% (w/v) sodium dodecyl sulphate (SDS), 150 mM ethylenediaminetetraaceticacid (EDTA), 0.4 mg/ml Proteinase K) and incubated at 40°C for 30 min. An equal volume of loading dye (95% (w/v) formamide, 20 mM EDTA, 0.05% (w/v) bromophenol blue and xylene cyanol FF, pH 8.0) was added and the samples were boiled at 95°C for 10 min followed by a rapid quench on ice for 3 min prior to loading on a TBE–urea gel (6% (w/v) acrylamide, 7 M urea, 90 mM Tris–borate, 2 mM EDTA). dsDNA markers for the mini-gels (GeneRuler 50 bp DNA ladder, Thermo Fisher Scientific) or for the sequencing gels (25 bp DNA ladder, Invitrogen), were end-labelled using T4 polynucleotide kinase and γ-^32^P-ATP (3000 Ci/mmol) and unincorporated nucleotides removed using Micro Bio-Spin 6 size-exclusion columns (Bio-Rad). Markers were diluted 10-fold with Reaction Buffer and prepared for electrophoresis as above for the transcription reactions.

Sequencing gels (17 × 40 × 0.04 cm, SequiGen system, Bio-Rad) were heated to 55°C by pre-running at 65 W (max 2 kV) for 1 h, whereas mini-gels (8.3 × 7.0 × 0.075 cm, Mini-Protean, Bio-Rad) were pre-run at 200 V for 1 h without temperature control. All gels were run in 90 mM Tris–borate, pH 8.0, 2 mM EDTA, with temperature control at 55°C for sequencing gels and without temperature control at room temperature for mini-gels. Following electrophoresis, gels were fixed for 30 min in 10% (v/v) methanol, 10% (v/v) acetic acid. Dried gels were exposed to a storage phosphor screen (Fujifilm), which was scanned using a Typhoon phosphorimager (GE).

### Data analysis

**Figure 2.**
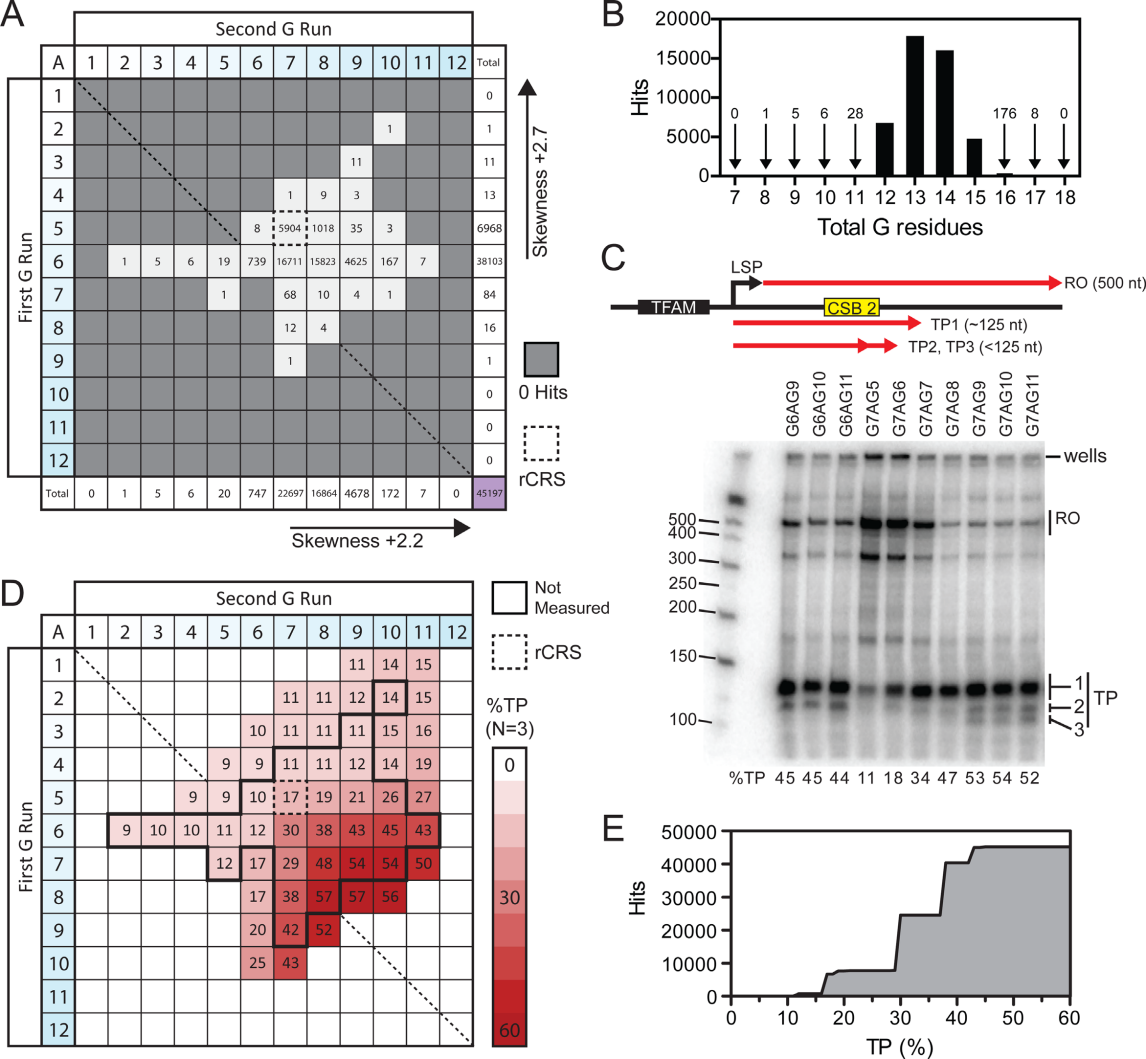
Distribution of length heterogeneity in sequenced NCRs of human mtDNA and the effect on the levels of POLRMT transcription termination. (**A**) Distribution of adenine-interrupted discontinuous CSB 2 G-tract hits from searches of complete and partial human mtDNA sequences deposited in GenBank (total of 45 197). (**B**) Number of hits from panel A summed according to total number of guanine residues. (**C**) *In vitro* transcription assay. The top panel cartoon shows the linear DNA substrate and the sizes of the run off (RO) and the three transcription products (TP1, TP2 or TP3) that can appear due to CSB 2 (yellow box). The black box represents the TFAM binding site upstream of the light strand promoter (LSP). The lower panel shows a representative mini-gel with the quantified percentage of total TP shown below each lane. The bands at the top of the gel are material retained in the wells. (**D**) The average percentage of total TP (TP1 + TP2 + TP3) measured for different CSB 2 sequences (*N =* 3). The black lines show the extent of sequences observed in panel A. Representative transcription mini-gels of all variants and bar graphs of the quantified data are shown in Supplementary Figures S5 and S6. E. Cumulative correlation plot of occupied variants from panel A and average TP percentages for those variants from panel D. Most of the occupied variants produced a total TP *in vitro* between 17% and 43%.

The 16-bit densitometric scans from the phosoimager were analysed using the 1D gel analysis software of ImageQuant (GE). All data graphs were produced and analysed using GraphPad Prism (GraphPad Software, Inc). For the scanned data from mini-gels, the density of lane regions corresponding to CSB II-dependent transcription termination products TP1, TP2, TP3 or TP (e.g. see Figure 2C) were used to calculate termination efficiency as a percentage of total lane density from the well to the ∼100 nt marker. The mean and standard deviations were calculated from three independent repeats. For the scanned data from sequencing gels, pixel position was converted to DNA length using the parameters from a first order Lagrange curve fitted to the 25 bp DNA ladder data. The density value of each pixel within a lane was then normalized to the maximum pixel density within the lane region that covered TP1-3 (92–140 nt). Skewness in Figure 2A was calculated using the SKEW function of Microsoft Excel.

## RESULTS

### Length heterogeneity of CSB 2 in sequenced human mitochondrial genomes

To evaluate the sequence variation at CSB 2 in human mitochondrial genomes, we examined sequences deposited in GenBank. We compiled a database based on both complete and partial human mtDNA sequences that included the NCR, and analysed the data using nBLAST (48,49). We first examined discontinuous sequences. We varied the number of guanines in each search string while keeping the central residue as adenine (variations in the identity of this nucleotide are considered in a later section). Our search algorithm had a minimal text string size of 25 nucleotides, so we used the rCRS up- and downstream of the G-tracts. We used the nomenclature G_m_AG_n_ to classify our sequences, where *m* and *n* are the number of guanines in the first and second G-runs, respectively. Note that this is the reverse complement of the polyC-T-polyC sequence often quoted in haplogroup studies. The number of sequences found for each variant are presented in matrix form in Figure 2A (45 197 sequences).

The majority of hits (>95%) cluster within a subset of sequences - the rCRS variant G5AG7 (13%), G6AG7 (37%), G6AG8 (35%) and G6AG9 (10%) (Figure 2A). We note that whilst the total length of these sequences varies between 12 and 15 guanines (Figure 2B), the first G-run is always shorter than the second G-run. Many variants are very low frequency (fewer than 20 hits). Some of these could be false hits due to problems in accurate sequencing of G-runs. However, we also note that other studies have reported CSB 2 sequences that lie outside of the distributions seen here—e.g. G6AG15, (37,43)—although these longer variants of the second G-run tend to always be found in studies of hair cells. The data from these studies are not always deposited and are thus not part of the database.

In most cases, variation occurs by changes in the length of just one of the two runs—i.e. most sequences occupy either the column or the row in Figure 2A that bisect the highest frequency sequence G6AG7. The spread around the G6AG7 variant is not symmetrical; in Figure 2A, >99% of sequences occupy the top-right quadrant while the bottom-left quadrant is under-occupied. This asymmetry is also reported by the skewness values for the row and column totals (Figure 2A). Overall, length heterogeneity appears to disfavour first G-runs longer than six residues, in particular when combined with second G-runs that are shorter than five residues.

### The effect of length heterogeneity on termination of POLRMT transcription

To assess how changes in the lengths of the first and second G-runs of discontinuous CSB 2 sequences affect POLRMT activity, we reconstituted transcription initiating from the LSP *in vitro* using recombinant proteins and DNA. We produced a library of DNA substrates that contained a region of the rCRS encompassing the LSP and CSB 3, 2 and 1 (nt 16 478-509) in which the number of guanine residues in CSB 2 were varied. We chose to analyse each of the ‘occupied variants’ found in our database search (Figure 2A) and, additionally, adjacent ‘unoccupied variants’ that differed by one or two nucleotides but which were not found in the database search. Templates were produced by PCR and mixed with POLRMT, TFAM and TFB2M under conditions that allowed multi-round transcription initiation from LSP (see Materials and Methods). Transcripts were labelled using incorporated radioactive UTP, and RNA products from a single time point were separated on denaturing acrylamide mini-gels. Transcription from the LSP to the end of the PCR template gave a run-off product (RO) of ∼500 nt (Figure 2C). Transcription products (TPs) that accumulate close to CSB 2 were ∼125 bp. TEFM was excluded to show the CSB 2-dependent product; the effect of TEFM is considered in a later section.

All of the CSB 2 variants were analysed in triplicate. An example transcription gel for a range of substrates is shown in Figure 2C. Representative gels for the complete data set are presented in Supplementary Figure S5. In all cases, we observed bands corresponding to the RO and TPs. Additional bands were also observed that represent intermediate/termination products at other sequences under our *in vitro* conditions. For all substrates, the TP region between ∼100–125 nt could be resolved into two distinct bands (TP1 and TP2, Figure 2C), similar to the premature termination products seen in other studies (5,6). With some substrates an additional shorter transcription product (TP3) could be resolved that has not been described previously (e.g. G7AG9, G7AG10 and G7AG11 Figure 2C). The band intensity of the total TP region (encompassing TP1, TP2 and TP3) relative to the complete lane (including other intermediates and the RO) was quantified for each substrate and is shown below the gel in Figure 2C (Materials and Methods). In the absence of a G-tract, stalling driven by the downstream poly-T tract alone is ∼9% (quantified in Figure 6). We consider TP values above this basal value as showing that the G-tract has increased termination at or close to CSB 2. Because of the quantitation method, the values here are considered as relative values to compare the effect of length heterogeneity, rather than absolute values.

The mean TP total percentages for the complete dataset are presented as a matrix heat map in Figure 2D. The quantified data and statistical variation are shown in Supplementary Figure S6. There was a general trend towards increased TP levels as the as the total length of the G-tract was increased, as one would predict from an increase in quadruplex stability as more guanine tetrads can form (52). In most cases only TP1 and TP2 bands were visible above background (Supplementary Figure S6B–D). The TP3 band became visible above background with substrates in the bottom-right quadrant as total TP levels increased to 43% or greater (Figure 2C, D and Supplementary Figure S6D). The formation of TP3 is considered in Supplementary Figure S7. Variants that could generate TP3 were only observed rarely in the database (20 out of 45 197), and this product is unlikely to be relevant *in vivo*.

Is there a correlation between the frequency of occupied variants found in the sequenced genome database (Figure 2A) and the corresponding TP levels observed *in vitro* (Figure 2D)? The majority of the occupied variants produced TP levels of 17–43% (Figure 2E). Variants with 8–11 guanines in total produced virtually no additional termination above the basal level and were correspondingly rarely found in the database (only 40 occupied variants, Figure 2B). Similarly, variants that produced high levels of termination (>43%) were rarely found in the database. The rCRS (G5AG7, Figure 1) produced a TP level half that of the two most common sequences, G6AG7 and G6AG8 (Figure 2D). However, similar TP levels were seen with unoccupied variants not found in the database (e.g. G7AG6, 17%), while even lower TP levels were observed in some occupied variants (e.g. G3AG9, 11%). It appears that TP levels by themselves cannot explain why some sequences are preferred over others.

**Figure 3.**
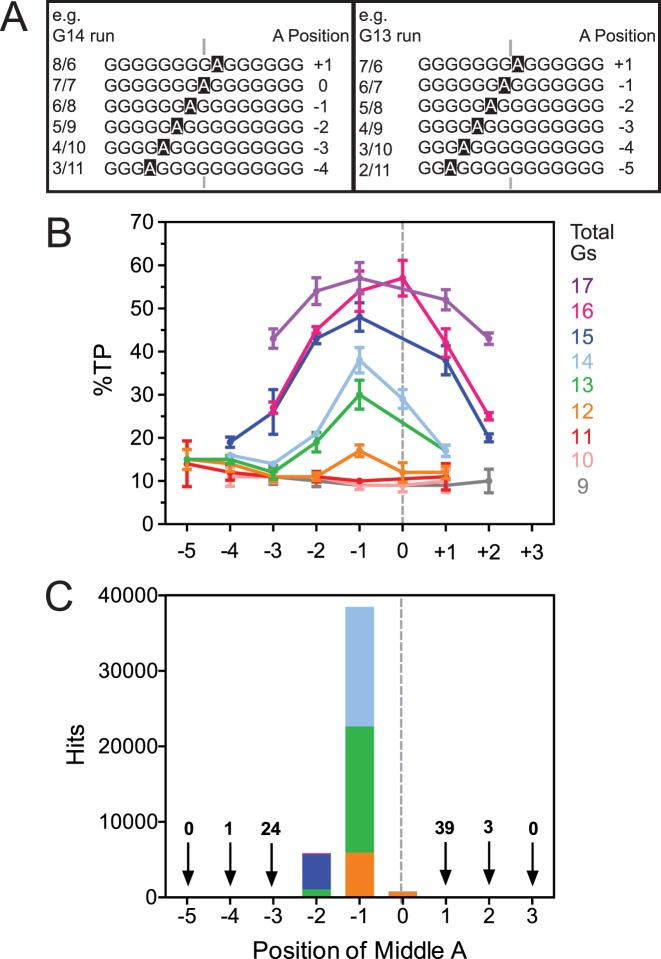
The influence of the position of the adenine of discontinuous CSB 2 sequences. (**A**) Definition of position of the middle adenine. Examples of G-tracts with even (G14) or odd (G13) numbers of guanines are shown. See text for full details. (**B**) Percentage of TP as a function of position of the adenine for discontinuous G-tracts of 9–17 residues. Data taken from Figure 2D and Supplementary Figure S6 (*N =* 3, error bars S.D.). (**C**) Data from Figure 2A plotted as a function of position of the adenine. Colours represent the occupied variants with G-tract lengths as in panel (B).

We noted that there was a marked asymmetry in the *in vitro* transcription data in Figure 2D, with the relative position of the adenine residue being crucial to the observed TP level rather than just the total number of guanines. This is most clearly shown by grouping the data from Figure 2D according to the total number of guanine residues and replotting the TP levels against the relative adenine position (Figure 3A and B). Where the first G-run was longer than the second G-run, the adenine was defined as having a positive position (i.e. towards the 3′ end of the G-tract). Where the first G-run was shorter than the second G-run, the adenine was defined as having a negative position (i.e. towards the 5′ end of the G-tract). For sequences with an even number of total guanine residues, there can also be a zero position where the lengths of the two runs are identical. For sequences with 11, 12, 13, 14, 15 or 17 guanines in total, the highest TP levels were observed with adenines at the −1 position. For the sequences with 16 guanines, the highest TP level was seen with the 0 position substrate.

Strikingly, ∼85% of the occupied variants from the sequenced genomes have the adenine at the −1 position (Figure 3C). Of these variants, almost all have 12–14 guanines, where a 5′ or 3′ shift in the adenine position from −1 would produce a reduction in TP levels (Figure 3A). Another ∼13% of the occupied variants have sequences with a −2 adenine. Of these, over three quarters have 15 guanines, where shifting from −1 to −2 produces only a small effect (Figure 3A). Therefore, the majority of genomic discontinuous CSB 2 sequences have a structure that favours the highest possible level of transcription termination given the length of the G-tract.

### The adenine residue that interrupts the G-tract of discontinuous CSB 2 sequences is not necessary for transcription termination

**Figure 4.**
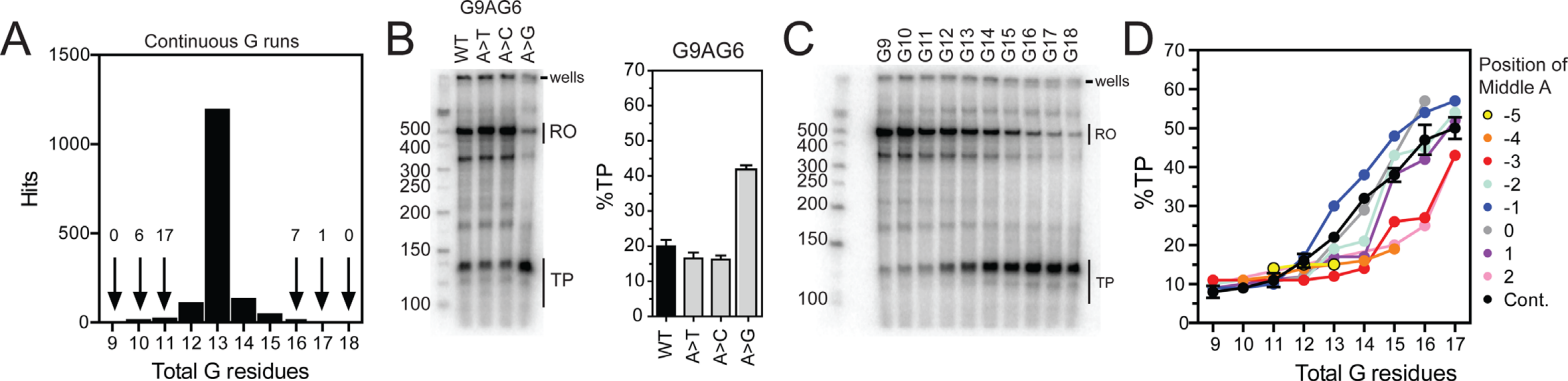
Comparison of discontinuous and continuous CSB 2 sequences. (**A**) Distribution of continuous CSB 2 G-tract hits from searches of complete and partial human mtDNA deposited in GenBank. (**B**) Representative mini-gel and the average percentage of total TP (*N =* 3, error bars S.D.) measured for the discontinuous CSB 2 sequences G9AG6 (WT), G9TG6 (A>T) and G9CG6 (A>C), and the continuous CSB 2 sequence G16 (A>G). (**C**) Representative mini-gel for transcription of discontinuous CSB 2 sequences G9 to G18. (**D**) The average percentage of total TP (*N =* 3, error bars S.D.) measured for the continuous CSB 2 sequences G9 to G18, plotted as a function of total number of guanines alongside the discontinuous CSB 2 data from Figure 2D and Supplementary Figure S6 grouped according to the position of the adenine as defined in the main text and Figure 3A. Error bars are omitted from the discontinuous data for clarity.

To investigate further the role of the interrupting adenine residue, we searched the human mtDNA sequences for CSB 2 variants where the adenine was replaced by either cytosine or thymine, or was missing altogether, producing a continuous G-tract. We found only two occurrences of a G_*m*_CG_*n*_ sequence [G10CG2 (53) and G5CG10 (54)] and only one occurrence of a G_*m*_TG_*n*_ sequence [G6TG8 (55)]. In contrast, continuous G-tracts were more frequent (1491 hits), in particular G13 (Figure 4A).

To determine how changes to the central adenine residue of CSB 2 affected transcription *in vitro*, we compared the TP levels using DNA substrates where the adenine was substituted with thymine, cytosine or guanine. We chose to modify the G9AG6 variant as the adenine is in a relatively inefficient +2 position (Figure 3B), allowing us to determine whether other bases enhanced or diminished formation of TPs. The G9AG6, G9TG6 and G9CG6 sequences all gave relatively similar TP levels (Figure 4B and C), suggesting that adenine, thymine and cytosine all have the same effect on transcription. In contrast, the G16 continuous sequence produced a more than two-fold increase in TP levels (Figure 4B).

The efficiency of TP formation by the continuous sequences G9 to G18 was then examined (Figure 4C); TP levels increased with G-tract length and only TP1 and TP2 were observed above background. The average quantified TP levels for the continuous sequences are compared to the values for the adenine-interrupted discontinuous sequences in Figure 4D. For total G-tract lengths of 14 residues or shorter, the continuous sequences were almost as efficient as the best discontinuous sequence (i.e. adenine at the −1 position). For total G-tract lengths of 15 residues or longer, there was less difference between the continuous G-tracts and the discontinuous sequences with adenine at −2, −1, 0 or +1. The presence of an interrupting base pair is thus not essential for efficient TP formation.

### The CSB 2 termination sites are not affected by length heterogeneity

To identify the 3′ termini of the RNA products that accumulate following transcription of CSB 2 and how these are affected by length heterogeneity, we separated the *in vitro* transcription reactions on larger denaturing ‘sequencing’ gels which give single nucleotide resolution (Supplementary Figures S8 and S9). The gels were scanned for band intensity and the data normalized to allow comparison of band positions (Materials and Methods).

**Figure 5.**
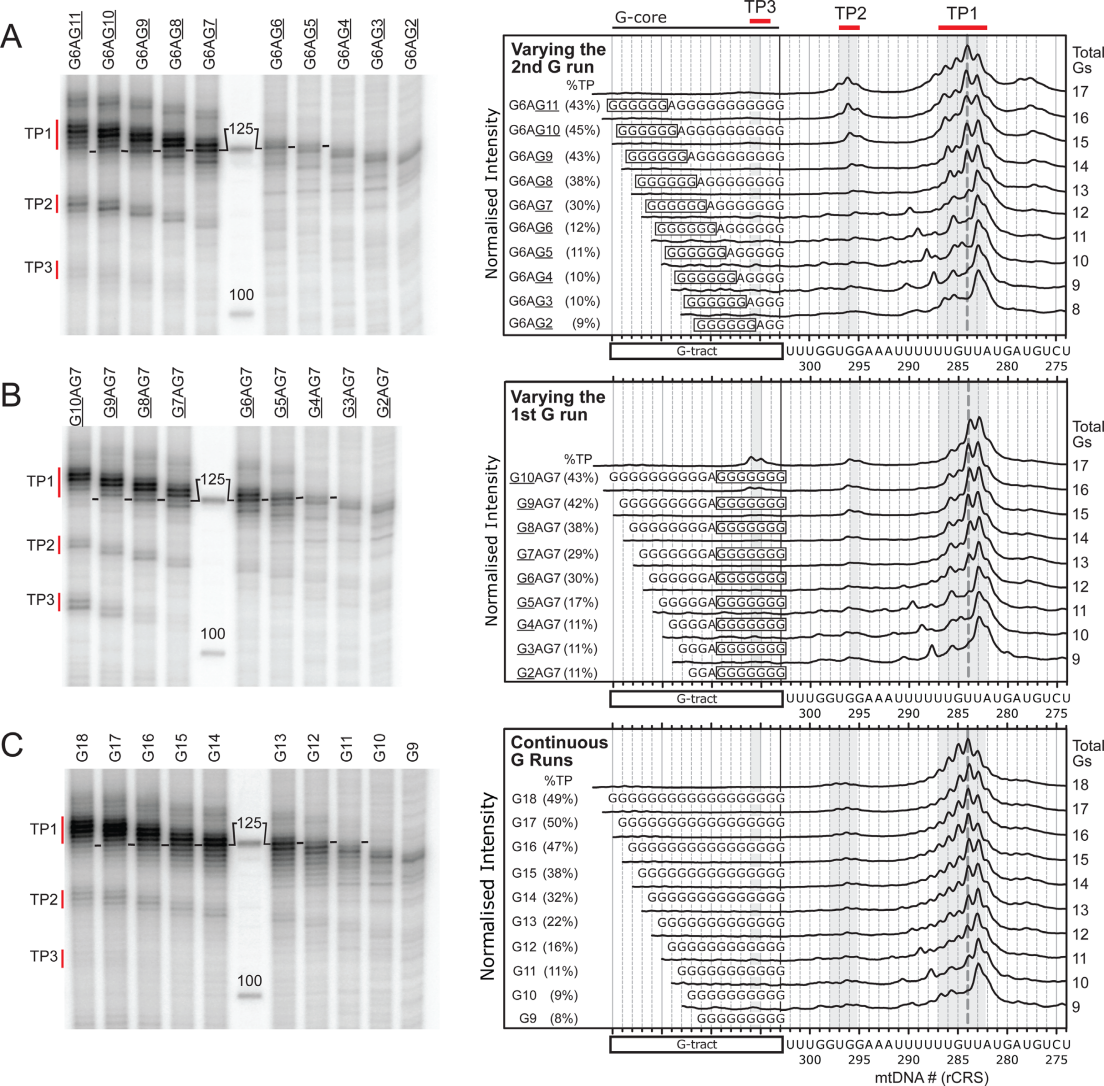
Mapping the locations of transcription products as a function of length heterogeneity. (**A**) Varying the length of the second G-run of discontinuous sequences. (*left panel*) Denaturing sequencing gel showing the TP products from transcription reactions on DNA substrates where the first G-run was fixed at six guanines and the second G-run was varied as shown (full gel in Supplementary Figure S9). Approximate positions of TP1, TP2 and TP3 are shown (note that these move for each variant, as explained in Supplementary Figure S8. (*right panel*) Lanes from the gel were scanned and processed as described in Materials and Methods to convert pixel position up the lane to a linear sequence scale (*x*-axis of graph) and to normalise the intensities so that the maximum peak heights in each lane are the same (arbitrary *y*-axis of graph). Total TP percentages are shown in the brackets from Figure 2D and Supplementary Figure S6 (*N =* 3, errors S.D.). To compare the positions of the TP bands, sequences were aligned using the 3′ terminal guanines of the G-tracts (black vertical line). Sequences downstream of the G-tract are numbered according to the rCRS (47) (Figure 1). The uracil corresponding to transcription of T_284_ is marked by the thick grey dotted line to help guide the eye. (**B**) Varying the length of the first G-run of discontinuous sequences. Transcription reactions on DNA where the second G-run was fixed at seven guanines and the first G-run was varied as shown. Data treated as in panel A. Full gel in Supplementary Figure S9. (**C**) Varying the length of continuous sequences. Transcription reactions on DNA where the length of continuous sequences was varied as shown. Data treated as in panel A except that total TP percentages are from Figure 4D (*N =* 3, errors S.D.). Full gel in Supplementary Figure S9.

Figure 5A shows a comparison of scanned data for discontinuous CSB 2 variants where the first G-run was kept constant at six residues (the most common value in Figure 2A) and the second G-run was varied between 2 and 11 residues. Residues downstream of the G-tracts were the same in all cases and are numbered according to the rCRS for ease of comparison (Figure 1) (47). Consistent with previous studies, the main termination products (encompassing TP1) were located immediately downstream of, and within, a T-tract (5′-T_291_TTTTT_286_-3′) (Figure 5A). For the shorter variants G6AG2 to G6AG6, which produced basal TP levels of 9–12%, the principal product terminated at T283. As the second G-run increased in length from 7 to 11 residues, further products were observed between T283–T288, with T284 eventually becoming the principal product. TP2 transcripts terminated at positions 5′-T_297_GG_295_-3′, and became more prominent as the total TP level increased.

Figure 5B shows a comparison of scanned data for discontinuous CSB 2 variants where the second G-run was kept constant at seven residues (the most common value—Figure 2A) and the first G-run was varied between 2 and 10 residues. Similar changes in the location of the TP products were observed as those described above, except that there were fewer products resulting from termination upstream of T_284_ and the TP2 products were less intense (Figure 5B).

Figure 5C shows a comparison of scanned data for continuous CSB 2 variants. For G9 to G11, which gave basal TP levels of ∼11%, the principal TP1 transcript terminated at T_283_. As the continuous sequences were extended from G12 to G18, a series of clearly-defined transcription products was observed between T_288_ to T_282_, with T_284_ becoming the principal product from G14 upwards. TP2 bands were less pronounced than with the discontinuous sequences, but mapped to a similar location.

For all the CSB 2 sequences investigated, TP1 and TP2 mapped to similar locations: the TP1 products in the region 287–282; and the TP2 products in the region 297–295. In both cases, there are T-tracts upstream of the 3′ ends of the transcription products that will produce runs of uracils in the transcript. Therefore, regardless of the identity of CSB2 or the level of termination produced, the 3′ ends of the transcripts terminate at similar locations which appear to be in proximity to T-tracts.

### Role for the DNA sequence downstream of the G-tract in transcription termination

**Figure 6.**
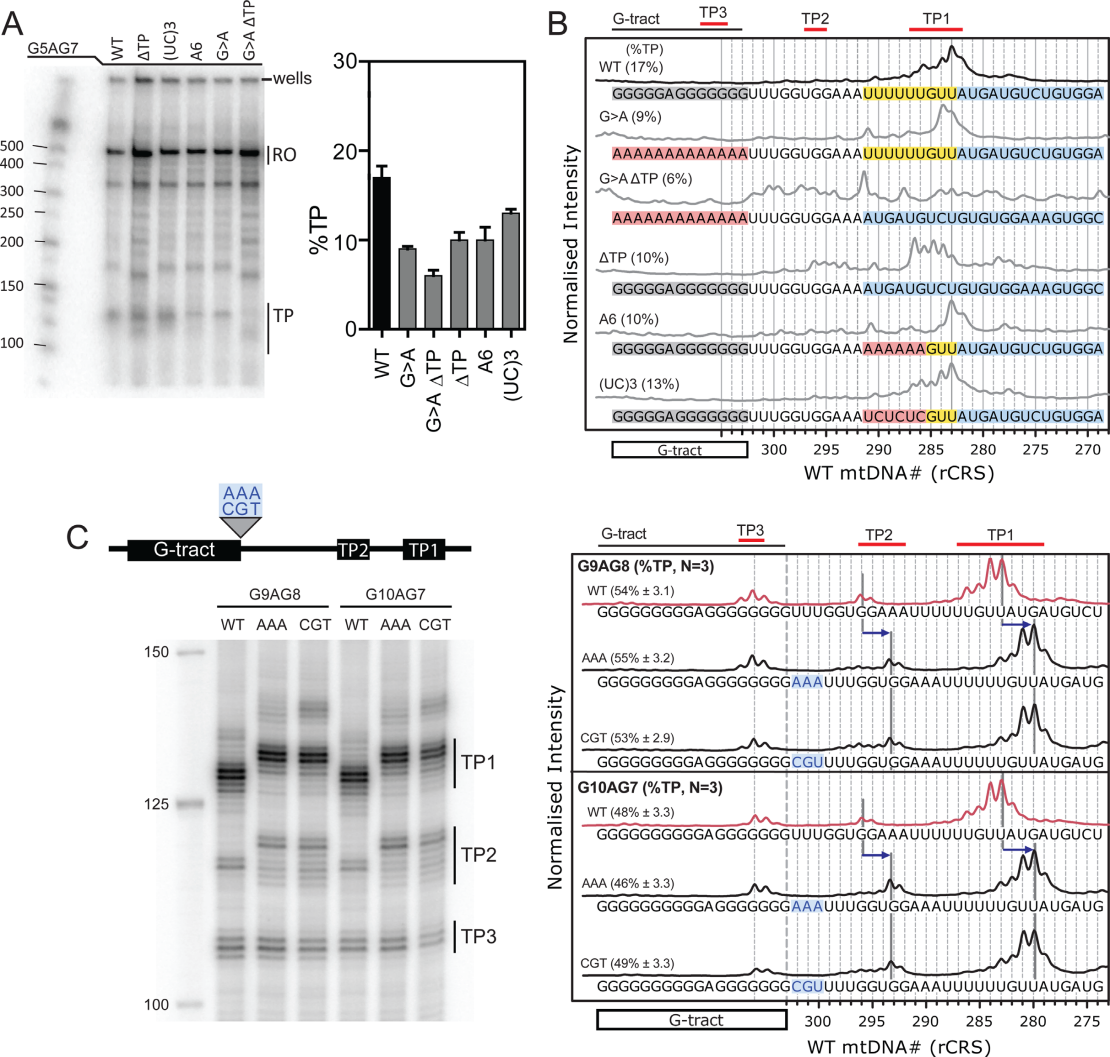
The role of sequences downstream of the CSB 2 G-tract in transcription termination. (**A**) Representative mini-gel and the average percentage of total TP (*N =* 3, error bars S.D.) measured for the discontinuous CSB 2 sequences G5AG7 (WT), and modifications to the G-tract (G>A) and/or the downstream sequences [TP, A6 or (UC)3]. Details of the sequence changes are shown in panel (B) and described in the main text. (**B**) Transcription reactions from panel A were also separated on a sequencing gel (Supplementary Figure S10) and the lanes scanned and processed as described in Figure 5. The 5′-T_291_TTTTTGTT_283_-3′ sequence is highlighted in yellow. Sequences changes are highlighted in red. The sequence downstream of the 5′-T_291_TTTTTGTT_283_-3′ sequence is highlighted in blue to emphasise that it moves relative to the G-core sequence in the ΔTP substrates. Percentages are the TP levels from panel (A) (*N =* 3, errors S.D.). (**C**) The effect of a three nucleotide insertion on the position of the transcription products. (*cartoon*) The CSB 2 variants G9AG8 and G10AG7 were mutated by insertion of AAA or CGU nucleotides 3′ to the G-tracts. Transcription reactions were separated on a sequencing gel (Supplementary Figure S10) and the lanes scanned and processed as described in Figure 5. Average percentages of total TP (*N =* 3, errors S.D.) were calculated from mini gels (Supplementary Figure S10). The positions of the main TP2 and TP1 bands are shown by grey lines, with the shift produced by the insertion mutations indicated by the blue arrows.

To examine the relative influence of the CSB 2 G-tract sequence versus the T-tract sequences on formation of the transcription products, we analysed a series of G5AG7 substrates where the G-tract and/or the downstream sequences were modified (Figure 6 and Supplementary Figure S10).

When the G-residues of G5AG7 were changed to adenines (G>A), then the TP levels were reduced from ∼17% to ∼9% (Figure 6A), but the principal products still mapped to T_283_/T_284_ (Figure 6B). We consider this to be the basal level of transcription termination driven by the T-tract alone for comparison with the data in Figure 2. We suggest that on this substrate the elongation complex is destabilized by the resulting poly rU•dA hybrid. Where we additionally deleted the 5′-T_291_TTTTTGTT_283_-3′ sequence (G>A ΔTP), TP levels reduced further to ∼6% (Figure 6A) and bands that could be resolved were more randomly-located (Figure 6B). We consider this to be non-specific background termination independent of either G-tract or T-tract sequences.

We then considered substrates where the G-tract region was present but the downstream sequence was altered. We deleted the 5′-T_291_TTTTTGTT_283_-3′ sequence alone (ΔTP) or mutated the 5′-T_291_TTTTT_286_-3′ sequence to to an A6-run (A6). In both cases the TP levels were reduced from ∼17% to ∼10% (Figure 6A). Where alternate thymines of the 5′-T_291_TTTTT_286_-3′ sequence were changed to cytosine to strengthen the hybrid in the elongation complex [(UC)_3_], the TP levels reduced more moderately from ∼17% to ∼13% (Figure 6A). For A6 and (UC)_3_, the principal transcription products mapped to the same relative location as for the WT sequence, ∼20 nt downstream of the G-tract (Figure 6B). For ΔTP a range of transcription products were observed 16–20 nt downstream of the G-tract (Figure 6B), and which may reflect the presence of uracils in the hybrid.

This data suggests that the levels and locations of transcription termination are due to the combined influence in the transcript of the runs of guanine and uracil residues, with polyU sequences acting as a termination signal in their own right. To explore this further we produced substrates with three nucleotides (AAA or CGT) inserted immediately downstream of either a G9AG8 or G10AG7 sequence (Figure 6C). Insertion of either spacer shifted the TP2 and TP1 bands by exactly three nucleotides downstream whilst the position of TP3 was unaffected. This shows that the location of the transcription products is not wholly due to a ‘molecular ruler’ starting at the G-tract. We instead propose that TP1 and TP2 are influenced by: (i) the G-tract, which causes varied product formation dependent upon its sequence, within a region covering at least 16–23 nt downstream independent of its sequence; and, (ii) the presence of thymine residues in the downstream region, in particular, but not exclusively, a poly T-run which both increases product formation and locates the 3′ ends more precisely.

### The transcription elongation factor TEFM reduces formation of all transcription termination products at CSB 2 independent of length heterogeneity

**Figure 7.**
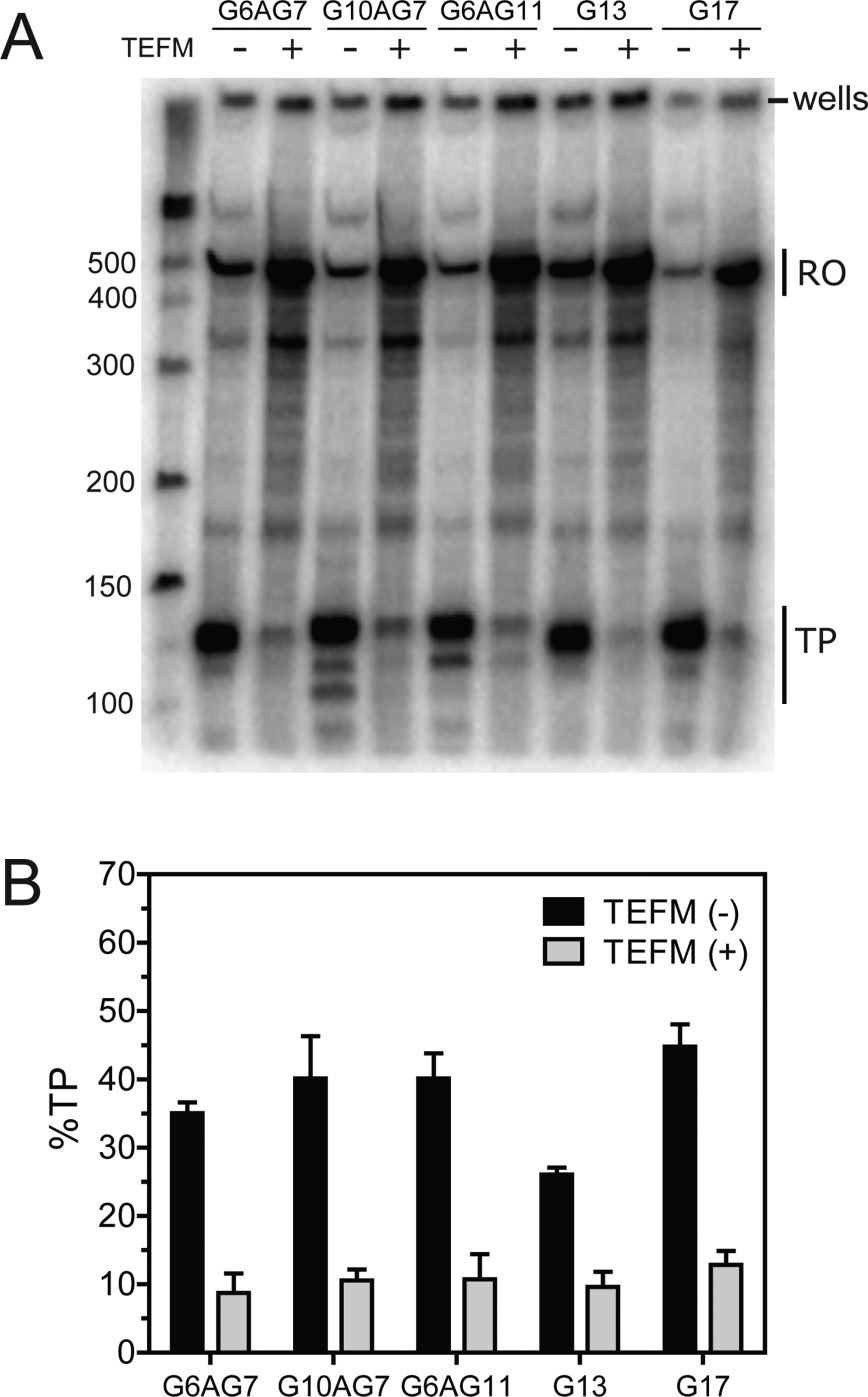
The influence of length heterogeneity on the anti-termination activity of the transcription factor TEFM. A representative mini-gel (**A**) and the average percentage of total TP (*N =* 3, error bars S.D.). (**B**) measured for the discontinuous CSB 2 sequences G6AG7, G10AG7 and G9AG11, and the continuous CSB 2 sequences G13 and G17, in the absence and presence of an equimolar concentration of TEFM dimer to POLRMT.

It has been demonstrated previously that TEFM can alleviate formation of transcription products within CSB 2 for both G5AG7 and G6AG8 sequences (22,23). Here we tested the effect of TEFM on *in vitro* transcription using a range of characteristic variants: G6AG7 (the most common sequence identified here); G10AG7 (a sequence that produces TP3 and which has a longer first G-run); G6AG11 (a sequence with a longer second G-run that produces high TP levels); and, G13 and G17 (continuous sequences producing intermediate and high TP levels, and which represent adenine deletion mutations of G6AG7 and G6AG11) (Figure 7). In all cases, TEFM reduced TP to close to basal levels seen in the absence of a G-tract (see Figure 6A). All transcription products were reduced, including the TP3 band seen with G10AG7. Therefore, TEFM can regulate POLRMT transcription product formation at CSB 2 independent of the exact sequence of the G-tract.

## DISCUSSION

It has been suggested previously that formation of a hybrid quadruplex at CSB 2 causes premature termination of transcription by POLRMT (5–7). From our analysis of the human mtDNA sequences, there is clear evidence that CSB 2 varies in the number of residues in both the first and second G-runs, although >84% of the changes are in the second G-run of a G6AG_n_ sequence, where *N =* 2–11 (Figure 2). As the total number of guanine residues was increased, there was a general trend towards increased transcription termination measured *in vitro* (Figure 2), as one would predict from an increase in quadruplex stability as more tetrads form (52)). However, regardless of the variant investigated *in vitro*, the 3′ ends of the terminated transcripts were located at similar downstream sequences (Figure 5). This is likely because of the influence of U-rich sequences in the transcript (Figure 6), consistent with the previous suggestion of a mechanism similar to rho-independent termination (22,23,56). We suggest that since variation in the number of guanine residues within CSB 2 produces changes in the amount of transcription termination without drastically changing the location of the products, length heterogeneity could be a mechanism to modulate the level of R-loop formation and consequently downstream events such as initiation of replication. TEFM was able to inhibit formation of all transcription products at CSB 2 regardless of the identity of the G-tract tested (Figure 7). Consequently, CSB 2 length heterogeneity would not necessarily result in a loss of control of transcription termination within the NCR. This also suggests that a mechanism to control TEFM activity would be required.

Our analysis of the deposited genome sequences revealed an asymmetric population distribution of discontinuous CSB 2 variants (Figure 2). The majority of occupied variants had the characteristic adenine in an off-centre location where the first G-run is one or two residues shorter than the second run. This trend correlates with the most efficient transcription product-forming sequences for a given number of guanines (Figure 3). When the adenine was placed closer to the 5′ or 3′ ends of the G-tract, formation of CSB 2-dependent transcription products was reduced. We suggest that in these CSB 2 sequences the adenine acts as a quadruplex-breaking residue and that these variants are avoided in the population by selection. This reduced efficiency may be because the position of the adenine produces a relatively short G-run at one or other end of the G-tract that cannot participate in quadruplex formation; the number of tetrads is instead limited by the length of the longer G-run. This effect is minimized or negated when the adenine is situated at a more central position where both G-runs are of sufficient length to participate in tetrad formation. The influence of the adenine position is less pronounced for longer discontinuous G-tracts, possibly because the longer G-run becomes equivalent to a more stable continuous G-tract (Figures 3 and 4). For short discontinuous sequences (9–11 guanine residues), where termination is near the basal level, there is little effect of the position of the adenine, possibly as quadruplex structures are not forming to any great extent.

Discontinuous CSB 2 sequences where the adenine is replaced with thymine or cytosine were virtually absent from the sequenced genomes, despite the fact that these residues produced the same quadruplex-breaking effect (Figure 4). Conversely, continuous sequences were more frequent relatively (Figure 4), were almost as efficient at transcription termination *in vitro* as the best discontinuous sequence (Figure 4), produced transcripts that mapped to the same downstream T-rich locations (Figure 5), and could be regulated by TEFM (Figure 7). Continuous sequences could arise due to mutation of the adenine residue by either transition or deletion, and are regularly reported as by-products of elevated length heterogeneity (28,41–44). The loss of the adenine residue would not necessarily alter the ability to control termination at CSB 2, and could in some instances remove an adenine from an unfavourable position. Nonetheless, the −1 discontinuous sequences remained the most efficient. When located at the −1 position, the adenine may favour formation of particularly stable quadruplex structures. However, it is unlikely that the adenine plays a special structural role. Instead the rarity of mutation of the adenine to thymine or cytosine most likely reflects the fact that transition mutation is a rare event compared to adenine deletion by elevated levels of polymerase slippage at CSB 2 (see below).

In addition to modulating quadruplex stability, CSB 2 length heterogeneity may result in increased heterogeneity in quadruplex structure (52). Longer sequences may also allow quadruplexes to seed in different registers; for example, there is evidence for an increased number of distinct transcription products upstream of T_284_ for the G12–G18 continuous sequences in Figure 5C. Notwithstanding the possible presence of a continuum of alternate structures, we observed that the transcription products always terminated close to the downstream T-rich sequences. This emphasises the importance of this genomic region as part of CSB 2. Because of the potential for heterogeneity in quadruplex structures resulting from length heterogeneity, we would argue that TEFM does not assist POLRMT by making specific interactions with a unique CSB 2 quadruplex structure, but instead through a general stabilization of the elongation complex that allows bypass of alternative structures.

If CSB 2 length heterogeneity allows for changes in the efficiency of transcription termination and stable R-loop formation, this may in turn allow for changes in the amount of initiation of DNA replication. Thus length heterogeneity could also influence mtDNA copy number, or alter D-loop structures that can play diverse roles in, for example, nucleoid localization or genome stability. What then causes hypervariation at CSB 2? Length changes in the G-tract are most likely due to slippage during replication (57). Genetic drift in the G-tract length may be driven by the mechanism of mutation during replication or alternatively by selective pressures that favour sequences that produce more transcription termination under conditions that require higher levels of mtDNA replication. As noted by Clayton (25,26), it is not clear why the second G-run should produce more replication slippage, and thus mutation, than the first G-run, or why other homopolymeric G-tracts found in mtDNA are not hypervariable. One answer might be that when mtDNA replication needs to be elevated, this requires more initiation events in the proximity of CSB2 and it is the initiation process itself that cause the mutations to arise principally in the second G-run. It is interesting to note that increased length heterogeneity has been reported in cells with elevated growth characteristics. Increased replication initiation could also lead to loss of the adenine by replication slippage, leading to the appearance of continuous sequences. The guanine-rich sequence of CSB 2 may be a hot spot for mutation by virtue of being a hot spot for replication initiation.

## Supplementary Material

SUPPLEMENTARY DATA
