# Supplementary material for: Length heterogeneity at conserved sequence block 2 in human mitochondrial DNA acts as a rheostat for RNA polymerase POLRMT activity
Source: Nucleic Acids Res. 2016 Jul 19;44(16):7817–29. doi: 10.1093/nar/gkw648 (PMC5027508; doi:10.1093/nar/gkw648)
Supplement: SUPPLEMENTARY DATA [file supp_44_16_7817__index.html]

Length heterogeneity at conserved sequence block 2 in human mitochondrial DNA acts as a rheostat for RNA polymerase POLRMT activity — Length heterogeneity at conserved sequence block 2 in human mitochondrial DNA acts as a rheostat for RNA polymerase POLRMT activity — SUPPLEMENTARY DATA 

# Length heterogeneity at conserved sequence block 2 in human mitochondrial DNA acts as a rheostat for RNA polymerase POLRMT activity

## SUPPLEMENTARY DATA

- SUPPLEMENTARY DATA
