# Supplementary material for: Length heterogeneity at conserved sequence block 2 in human mitochondrial DNA acts as a rheostat for RNA polymerase POLRMT activity
Source: Nucleic Acids Res. 2016 Jul 19;44(16):7817–29. doi: 10.1093/nar/gkw648 (PMC5027508; doi:10.1093/nar/gkw648)
Supplement: SUPPLEMENTARY DATA [file supp_gkw648_nar-01608-v-2016-File009.docx]

Supplementary Information to:

Length heterogeneity at conserved sequence block 2 in human mitochondrial DNA acts as a rheostat for RNA polymerase POLRMT activity

Benedict G. Tan, Frederick C. Wellesley, Nigel J. Savery* and Mark D. Szczelkun*

DNA-Protein Interactions Unit, School of Biochemistry, University of Bristol, Bristol, BS8 1TD, UK

**A.**

MGSSHHHHHHSQDPNSSSSASPQEQDQDRRKDWGHVELLEVLQARVRQLQAESVSEVVVNRVDVARLPECGSGDGSLQPPRKVQMGAKDATPVPCGRWAKILEKDKRTQQMRMQRLKAKLQMPFQSGEFKALTRRLQVEPRLLSKQMAGCLEDCTRQAPESPWEEQLAQLLQEAPGKLSLDVEQAPSGQHSQAQLSGQQQRLLAFFKCCLLTDQLPLAHHLLVVHHGQRQKRKLLTLDMYNAVMLGWARQGAFKELVYVLFMVKDAGLTPDLLSYAAALQCMGRQDQDAGTIERCLEQMSQEGLKLQALFTAVLLSEEDRATVLKAVHKVKPTFSLPPQLPPPVNTSKLLRDVYAKDGRVSYPKLHLPLKTLQCLFEKQLHMELASRVCVVSVEKPTLPSKEVKHARKTLKTLRDQWEKALCRALRETKNRLEREVYEGRFSLYPFLCLLDEREVVRMLLQVLQALPAQGESFTTLARELSARTFSRHVVQRQRVSGQVQALQNHYRKYLCLLASDAEVPEPCLPRQYWEALGAPEALREQPWPLPVQMELGKLLAEMLVQATQMPCSLDKPHHSSRLVPVLYHVYSFRNVQQIGILKPHPAYVQLLEKAAEPTLTFEAVDVPMLCPPLPWTSPHSGAFLLSPTKLMRTVEGATQHQELLETCPPTALHGALDALTQLGNCAWRVNGRVLDLVLQLFQAKGCPQLGVPAPPSEAPQPPEAHLPHSAAPARKAELRRELAHCQKVAREMHSLRAEALYRLSLAQHLRDRVFWLPHNMDFRGRTYPCPPHFNHLGSDVARALLEFAQGRPLGPHGLDWLKIHLVNLTGLKKREPLRKRLAFAEEVMDDILDSADQPLTGRKWWMGAEEPWQTLACCMEVANAVRASDPAAYVSHLPVHQDGSCNGLQHYAALGRDSVGAASVNLEPSDVPQDVYSGVAAQVEVFRRQDAQRGMRVAQVLEGFITRKVVKQTVMTVVYGVTRYGGRLQIEKRLRELSDFPQEFVWEASHYLVRQVFKSLQEMFSGTRAIQHWLTESARLISHMGSVVEWVTPLGVPVIQPYRLDSKVKQIGGGIQSITYTHNGDISRKPNTRKQKNGFPPNFIHSLDSSHMMLTALHCYRKGLTFVSVHDCYWTHAADVSVMNQVCREQFVRLHSEPILQDLSRFLVKRFCSEPQKILEASQLKETLQAVPKPGAFDLEQVKRSTYFFS

**B.**

MGSSHHHHHHSSGLVPRGSHMSSVLASCPKKPVSSYLRFSKEQLPIFKAQNPDAKTTELIRRIAQRWRELPDSKKKIYQDAYRAEWQVYKEEISRFKEQLTPSQIMSLEKEIMDKHLKRKAMTKKKELTLLGKPKRPRSAYNVYVAERFQEAKGDSPQEKLKTVKENWKNLSDSEKELYIQHAKEDETRYHNEMKSWEEQMIEVGRKDLLRRTIKKQRKYGAEEC

**C.**

MKSTTPKKITPNVTFCDENAKEPENALDKLFSSEQQASILHVLNTASTKELEAFRLLRGRRSINIVEHRENFGPFQNLESLMNVPLFKYKSTVQVCNSILCPKTGREKRKSPENRFLRKLLKPDIERERLKAVNSIISIVFGTRRIAWAHLDRKLTVLDWQQSDRWSLMRGIYSSSVYLEEISSIISKMPKADFYVLEKTGLSIQNSSLFPILLHFHIMEAMLYALLNKTFAQDGQHQVLSMNRNAVGKHFELMIGDSRTSGKELVKQFLFDSILKADPRVFFPSDKIVHYRQMFLSTELQRVEELYDSLLQAIAFYELAVFDSQPLEHHHHHH

**D.**

ATGAAATCTACGACTCCGAAGAAGATCACCCCGAATGTGACGTTCTGCGATGAGAATGCCAAAGAACCGGAAAACGCTTTAGACAAACTCTTTTCAAGCGAACAGCAGGCCAGTATTCTCCACGTTCTGAATACAGCATCGACCAAAGAACTGGAAGCGTTTCGCTTATTGCGCGGTCGTCGTAGCATCAACATTGTCGAACATCGTGAGAATTTCGGGCCTTTTCAGAACCTCGAATCGCTGATGAATGTCCCGCTGTTTAAATACAAGAGTACCGTGCAAGTTTGCAACTCCATTCTGTGTCCCAAAACTGGCCGCGAAAAGCGTAAGTCTCCGGAAAATCGCTTTCTGCGCAAACTGCTGAAACCCGACATTGAACGCGAACGTCTGAAAGCCGTAAACAGCATCATCAGTATTGTGTTCGGAACGCGCCGTATTGCTTGGGCACATTTAGACCGGAAACTGACGGTACTGGATTGGCAGCAAAGCGATCGCTGGAGTCTTATGCGTGGCATCTATAGTAGCTCGGTGTATCTGGAGGAGATTTCCTCGATCATTTCCAAAATGCCGAAAGCAGACTTCTATGTACTGGAGAAAACCGGTTTGTCCATTCAGAACTCCAGCCTGTTTCCGATTCTTCTGCATTTTCACATCATGGAAGCGATGCTGTATGCGTTGCTGAACAAAACCTTTGCGCAGGATGGGCAACATCAGGTCCTTTCGATGAACCGGAATGCGGTTGGCAAACACTTCGAGCTTATGATTGGTGATTCACGCACATCAGGCAAAGAGCTGGTGAAACAGTTCCTGTTTGATAGCATCTTAAAGGCTGATCCACGTGTTTTCTTTCCTAGCGATAAAATCGTGCATTATCGCCAGATGTTCTTATCAACCGAACTGCAACGCGTGGAAGAACTGTACGATTCTCTCTTGCAAGCAATTGCGTTCTACGAATTGGCCGTCTTTGACTCTCAGCCACTCGAGCACCACCACCACCACCACTGA

**Supplementary Figure S1: DNA and Protein Sequences.**

**A.** Protein sequence of human POLRMT with mitochondrial signal peptide removed (aa 41-1230) and a N-terminal His_6_ tag. Additional amino acids are highlighted in grey. **B.** Protein sequence of human TFAM with mitochondrial signal peptide removed (aa 43-246) and a N-terminal His_6_ tag. Additional amino acids are highlighted in grey. **C.** Protein sequence of human TEFM with mitochondrial signal peptide removed (aa 36-360) and a C-terminal His_6_ tag. Additional amino acids are highlighted in grey. **D.** *Escherichia coli* codon-optimised hTEFM gene sequence in pET24b-TEFM.

**Insertion of SpeI site into KpnI site**

placSpe1 CGGATAACTAGTTGGAGCGTAC

placSpe2 GCTCCAACTAGTTATCCGGTAC

**NCR primers with Spe1 sites**

NCRSpeF GCATGACTAGTCACCAGTCTTGTAAACC

NCRSpeR CCTAGACTAGTCTAAGAGCTAATAG

**DM of RPE1 mtDNA to make pGC-NCR(rCRS)**

C2301Ts GATGTCTGTGTGGAAAGTGGCTGTGCAGACATTCA

C2301Tas TGAATGTCTGCACAGCCACTTTCCACACAGACATC

G2412As TGAACGTAGGTGCGATAAATAATAGGATGAGGCAGG

G2412Aas CCTGCCTCATCCTATTATTTATCGCACCTACGTTCA

G2614As GGCTATTTAGGCTTTATGACCCTGAAGTAGGAACC

G2614Aas GGTTCCTACTTCAGGGTCATAAAGCCTAAATAGCC

A2941Gs ACTTGCTTGTAAGCATGGGGAGGGGGTTTTGATGT

A2941Gas ACATCAAAACCCCCTCCCCATGCTTACAAGCAAGT

del2252s CAGAAGCGGGGGAGGGGGGGTTT

del2252as AAACCCCCCCTCCCCCGCTTCTG

**Supplementary Figure S2: Primers pairs used for cloning of NCR of the human mtDNA from RPE1 cells and the subsequent site-directed mutagenesis to produce the rCRS.**

Numbers in the primer names indicate the position on the RPE1 mtDNA rather than the numbering of the rCRS.

A B

C

CSB2nXF TTTGGTGGAAATTTTTTGTTATGATGTCTG

CSB2n1F AGTTTGGTGGAAATTTTTTGTTATGATGTCTG

CSB2n2F AGGTTTGGTGGAAATTTTTTGTTATGATGTCTG

CSB2n3F AGGGTTTGGTGGAAATTTTTTGTTATGATGTCTG

CSB2n4F AGGGGTTTGGTGGAAATTTTTTGTTATGATGTCTG

CSB2n5F AGGGGGTTTGGTGGAAATTTTTTGTTATGATGTCTG

CSB2n6F AGGGGGGTTTGGTGGAAATTTTTTGTTATGATGTCTG

CSB2n7F AGGGGGGGTTTGGTGGAAATTTTTTGTTATGATGTCTG

CSB2n8F AGGGGGGGGTTTGGTGGAAATTTTTTGTTATGATGTCTG

CSB2n9F AGGGGGGGGGTTTGGTGGAAATTTTTTGTTATGATGTCTG

CSB2n10F AGGGGGGGGGGTTTGGTGGAAATTTTTTGTTATGATGTCTG

CSB2n11F AGGGGGGGGGGGTTTGGTGGAAATTTTTTGTTATGATGTCTG

CSB2n12F AGGGGGGGGGGGGTTTGGTGGAAATTTTTTGTTATGATGTCTG

CSB2m0R GCTTCTGGCCACAGCACTTAAACACATC

CSB2m1R CGCTTCTGGCCACAGCACTTAAACACATC

CSB2m2R CCGCTTCTGGCCACAGCACTTAAACACATC

CSB2m3R CCCGCTTCTGGCCACAGCACTTAAACACATC

CSB2m4R CCCCGCTTCTGGCCACAGCACTTAAACACATC

CSB2m5R CCCCCGCTTCTGGCCACAGCACTTAAACACATC

CSB2m6R CCCCCCGCTTCTGGCCACAGCACTTAAACACATC

CSB2m7R CCCCCCCGCTTCTGGCCACAGCACTTAAACACATC

CSB2m8R CCCCCCCCGCTTCTGGCCACAGCACTTAAACACATC

CSB2m9R CCCCCCCCCGCTTCTGGCCACAGCACTTAAACACATC

CSB2m10R CCCCCCCCCCGCTTCTGGCCACAGCACTTAAACACATC

CSB2m11R CCCCCCCCCCCGCTTCTGGCCACAGCACTTAAACACATC

CSB2m12R CCCCCCCCCCCCGCTTCTGGCCACAGCACTTAAACACATC

CSB2m13R CCCCCCCCCCCCCGCTTCTGGCCACAGCACTTAAACACATC

CSB2m14R CCCCCCCCCCCCCCGCTTCTGGCCACAGCACTTAAACACATC

CSB2m15R CCCCCCCCCCCCCCCGCTTCTGGCCACAGCACTTAAACACATC

CSB2m16R CCCCCCCCCCCCCCCCGCTTCTGGCCACAGCACTTAAACACATC

CSB2m17R CCCCCCCCCCCCCCCCCGCTTCTGGCCACAGCACTTAAACACATC

CSB2m18R CCCCCCCCCCCCCCCCCCGCTTCTGGCCACAGCACTTAAACACATC

**Supplementary Figure S3:** Primers pair combinations used for site-directed mutagenesis (SDM).

Primers pairs used to produce the adenine-interrupted discontinuous (**A**) CSB2 variants or continuous CSB2 variants (**B**). Primer sequences in **C**.

**CSB2 G>A**

CSB2G>AF AAAAAAATTTGGTGGAAATTTTTTGTTATGATGTCTG

CSB2G>AR TTTTTTGCTTCTGGCCACAGCACTTAAACACATC

**CSB2 ΔTP1**

TP1delF ATGATGTCTGTGTGGAAAGTGGCTGTGCAG

TPMutR TTTCCACCAAACCCCCCCTCCCCCG

**CSB2 G>A ΔTP1**

TP1delF ATGATGTCTGTGTGGAAAGTGGCTGTGCAG

CSB2G>AR2 TTTCCACCAAATTTTTTTTTTTTTGCTTCTGGCCACAGCAC

**TP1 mutant (UC)3**

TP1UC3F TCTCTCGTTATGATGTCTGTGTGGAAAGTGGCTGTGCAG

TPMutR TTTCCACCAAACCCCCCCTCCCCCG

**TP1 mutant A6**

TP1MutA6F AAAAAAGTTATGATGTCTGTGTGGAAAGTGGCTGTGCAG

TPMutR TTTCCACCAAACCCCCCCTCCCCCG

**G9AG8 spacer AAA spacer**

aaaF AAATTTGGTGGAAATTTTTTGTTATGATGTCTGTGTGGAAAGTGG

G9AG8R CCCCCCCCTCCCCCCCCCGCTTCTGGCCACAGCAC

**G9AG8 spacer CGT spacer**

cgtF CGTTTTGGTGGAAATTTTTTGTTATGATGTCTGTGTGGAAAGTGG

G9AG8R CCCCCCCCTCCCCCCCCCGCTTCTGGCCACAGCAC

**G10AG7 spacer AAA spacer**

aaaF AAATTTGGTGGAAATTTTTTGTTATGATGTCTGTGTGGAAAGTGG

G10AG7R CCCCCCCTCCCCCCCCCCGCTTCTGGCCACAGCAC

**G10AG7 spacer CGT spacer**

cgtF CGTTTTGGTGGAAATTTTTTGTTATGATGTCTGTGTGGAAAGTGG

G10AG7R CCCCCCCTCCCCCCCCCCGCTTCTGGCCACAGCAC

**G9AG6 Middle A mutation A>C**

G9CG6F CGGGGGGTTTGGTGGAAATTTTTTGTTATGATGTCTG

CSB2n9R CCCCCCCCCGCTTCTGGCCACAGCACTTAAACACATC

**G9AG6 Middle A mutation A>T**

G9TG6F TGGGGGGTTTGGTGGAAATTTTTTGTTATGATGTCTG

CSB2n9R CCCCCCCCCGCTTCTGGCCACAGCACTTAAACACATC

**Supplementary Figure S4: Primers pair combinations used for site-directed mutagenesis to produce the CSB2 variants and downstream mutants as indicated.**


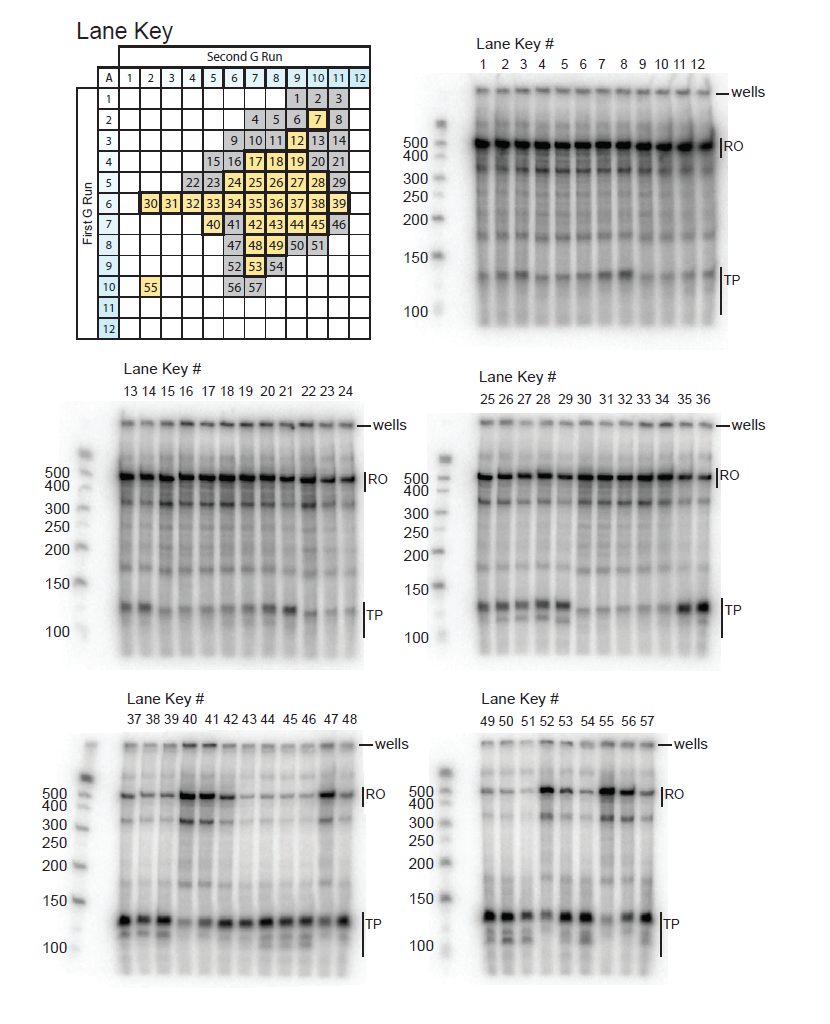


**Supplementary Figure S5: Transcription assays of CSB 2 variants.**

Representative mini-gels from *in vitro* transcription assays using the CSB 2 variants as indicated by the key. The G10AG2 substrate (lane key #55) was originally included in our *in vitro* mini gel assays but was subsequently excluded from further analysis.


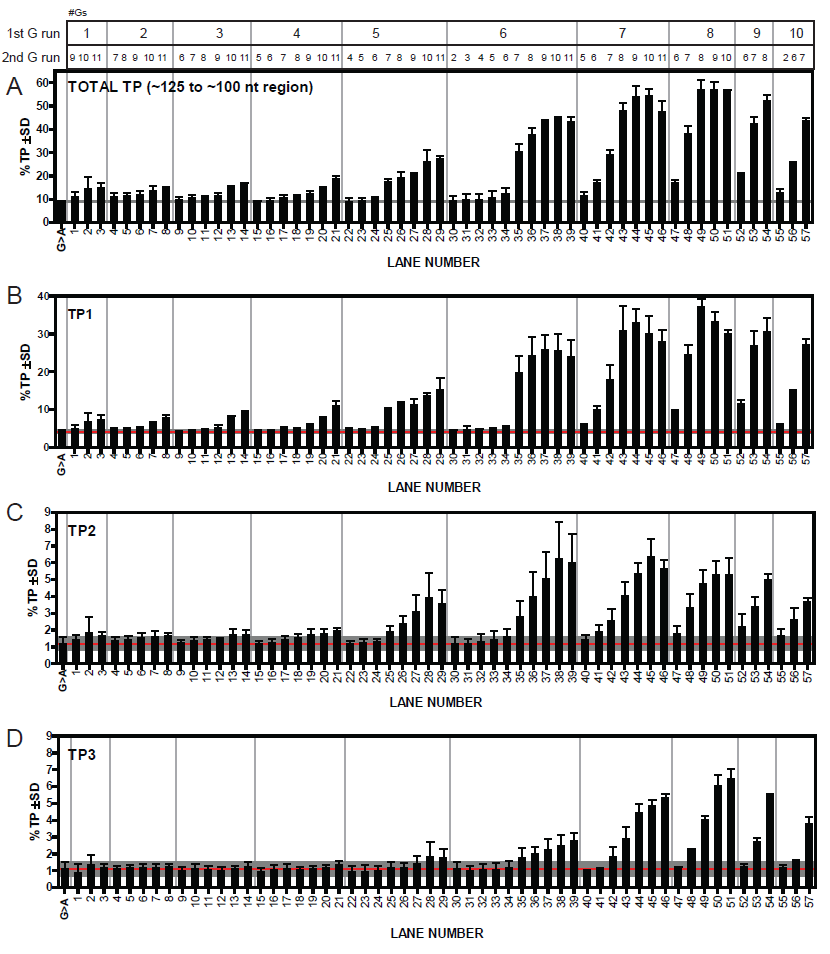


**Supplementary Figure S6: Quantification of the effect of length heterogeneity on transcription product levels.**

**A.** Total TP levels measured from transcription assays using the CSB 2 variants quantified from mini-gels as indicated by the key in **Supplementary Figure S5** (*N =* 3, error bars S.D.). The red line represents a basal TP level of (grey box are the S.D.), as measured in the absence of quadruplex formation where stalling is driven by the downstream poly-T tract alone (see G>A substrate in **Figure 6**). **B-D.** TP1, TP2 and TP3 levels, as indicated, measured separately from transcription assays using the CSB 2 variants as indicated by the key in **Supplementary Figure S5** (*N =* 3, error bars S.D.). The red lines represents basal TP levels (grey boxes are the S.D.), as measured in the absence of quadruplex formation where termination is driven solely by the downstream poly-T tract (**Figure 6**). Note the different *y*-axes scales.


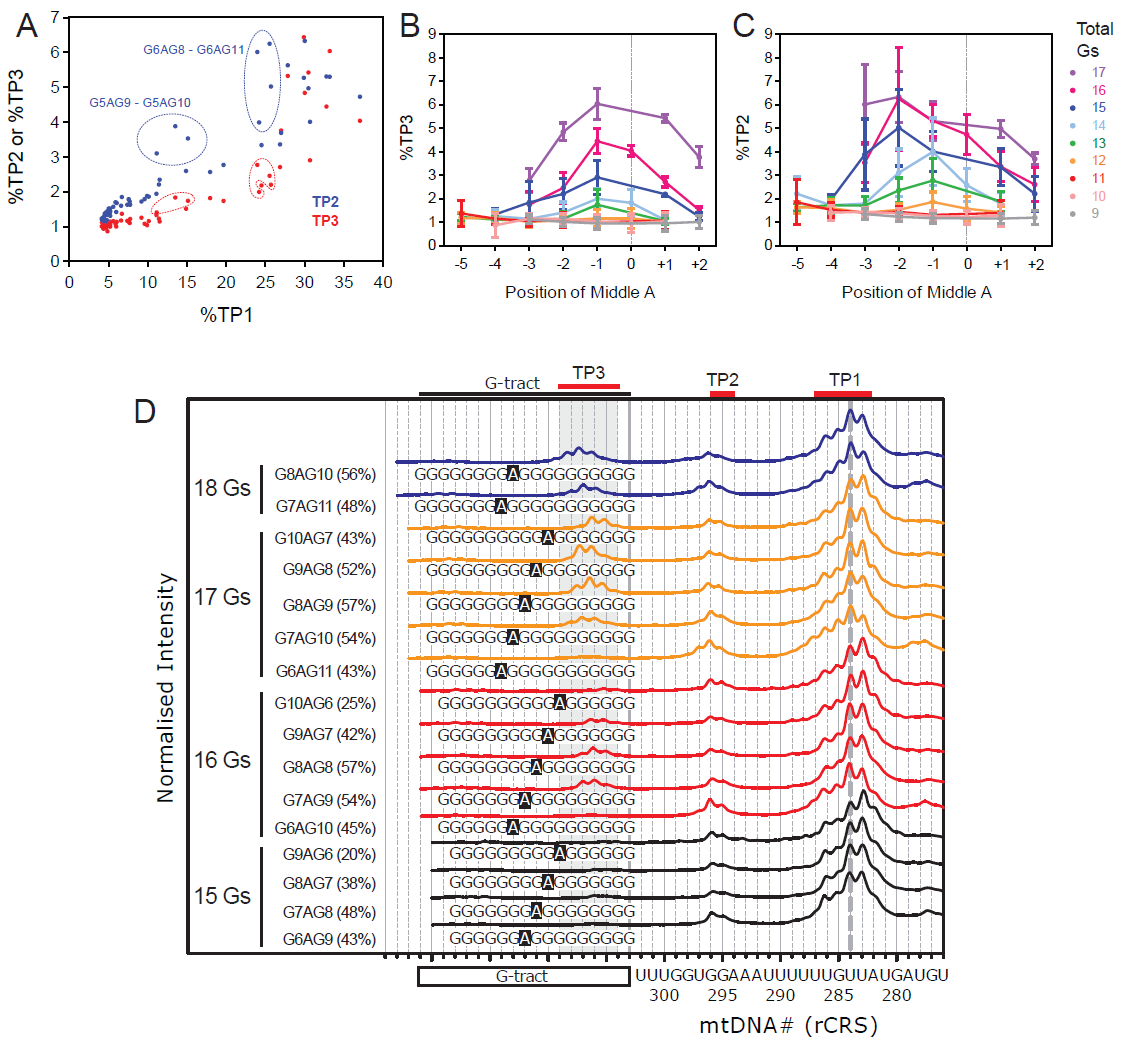


**Supplementary Figure S7: TP3 products are dependent on elongated, discontinuous G-tracts and are located within the second G-run.**

**A.** Plot of TP1 levels versus with TP2 or TP3 levels on the same CSB 2 variant (data from **Supplementary Figure S6B-D**). The dotted shapes indicate two groups of variants (G5AG9-G5AG11 and G6AG8-G6AG11) where there is a marked increase in the amount of TP2 produced relative to TP3. The appearance of the TP3 products above 3% appears to correlate with longer discontinuous CSB 2 variants of 16-18 guanines that in turn produce relatively high levels of total TP1. In comparison, TP2 products above 3% were observed with shorter discontinuous sequences (as low as 14 guanines). **B-C.** Percentages of TP3 (panel B) or TP2 (Panel C) as a function of position of the adenine (defined in **Figure 3A**) for discontinuous G-tracts of 9 to 17 residues. Data taken from **Supplementary Figure S6C, D** (*N =* 3, error bars S.D.). Maximum TP3 formation for the 15-17 guanine sequences required that the adenine was located centrally at -1. Given that continuous sequences do not produce TP3 above background regardless of length (**Figure 4 and 5**), it appears that this product may absolutely require the adenine. For TP2 products there is a less clear relationship with the adenine position since the data error is more significant. Since TP2 bands were also observed with continuous sequences (**Figure 5C**), the adenine is not absolutely required to generate this product. **C.** Mapping the position of TP3. Scanned and normalised data from the sequencing gels in **Supplementary Figure S9 and S11** is shown alongside data from **Figure 5**. As in **Figure 5**, the data was aligned to the 3ʹ terminal guanine of the G-tracts. The data is grouped according to the total number of guanines. Numbers in brackets are the average TP percentages from **Figure 2D and Supplementary Figure S6A**. For all sequences the positions of the TP1 and TP2 products align closely. The positions of the main TP3 bands were located 3-6 bp 5ʹ to the end of the G-tract. However, the rules governing the exact location of the main band are not clear, since they vary with both adenine position and number of guanines.


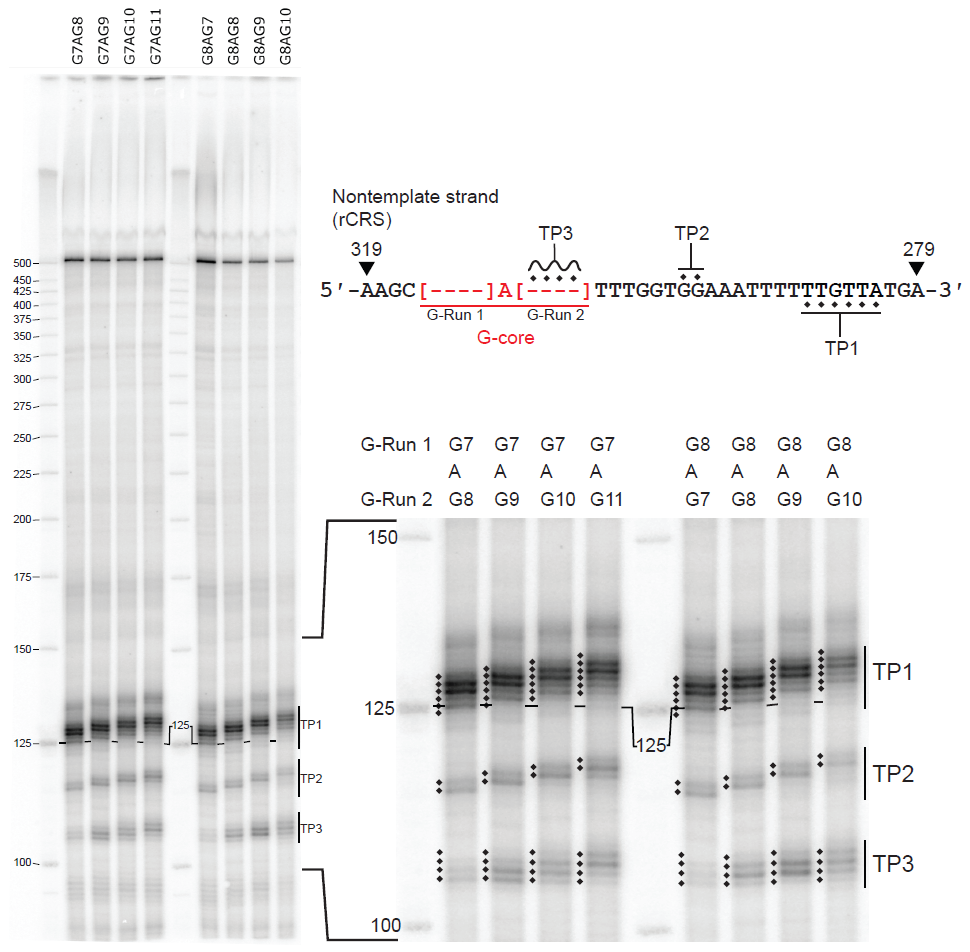


**Supplementary Figure S8: Separation of CSB 2 transcription products using a sequencing gel.**

A full sequencing gel (*left*) and a magnified portion showing the TP region (*right*) for products from transcription reactions on DNA where the first G-run was fixed at either seven or eight guanines and the second G-run varied as indicated. Approximate positions of TP1, TP2 and TP3 are indicated. The rCRS ^47^ is shown, with the variable G-tract in red. Because length heterogeneity causes a change in distance between the transcription start site and the sequences downstream of the G-tract, transcription products that terminate at the same site on different substrates can have different lengths. For example, because TP1 and TP2 map to regions downstream of the G-tract, as the length of the second G-run increases, these bands move up the gel (i.e., the transcripts become longer). This is shown by the diamonds symbols which represent the sequences T_287_TGTTA_282_ (TP1) and G_296_G_295_ (TP2) of the rCRS. The TP3 bands map to the second G-run and are more static (for example, relative to the 100 nt marker which aligns with the first G-run). Because of these relative differences in size of the products, in **Figures 5, 6 and 7** we scanned the intensity of each sequencing lane and aligned the data to the final guanine of the G-tract, allowing direct comparison of the locations of the downstream products.


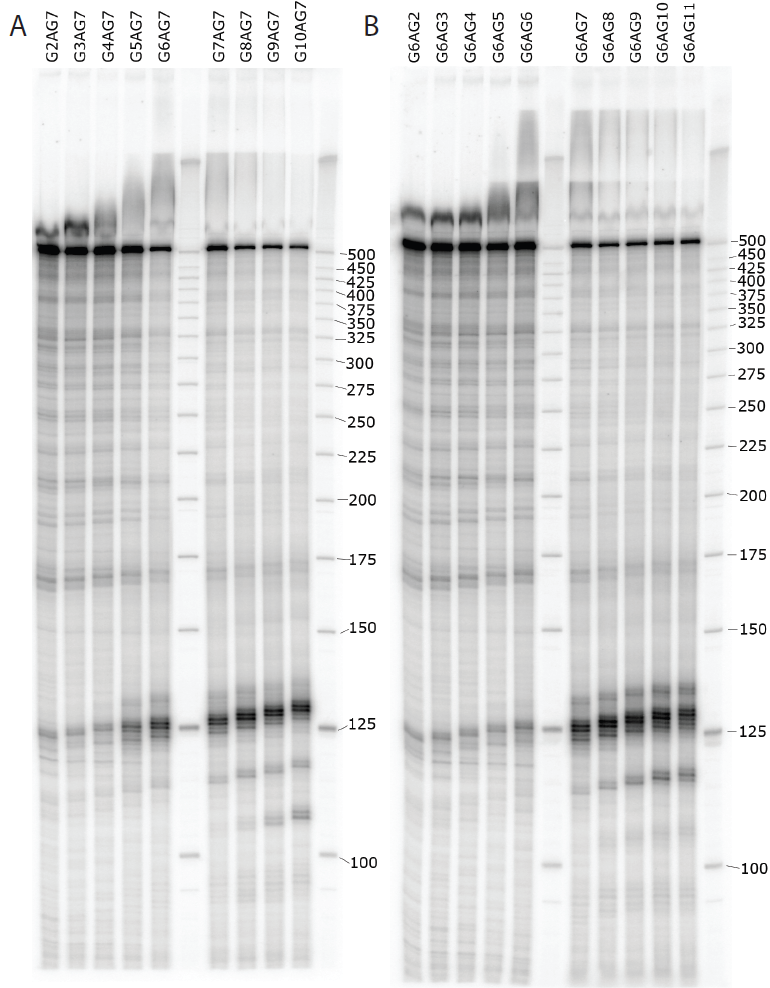


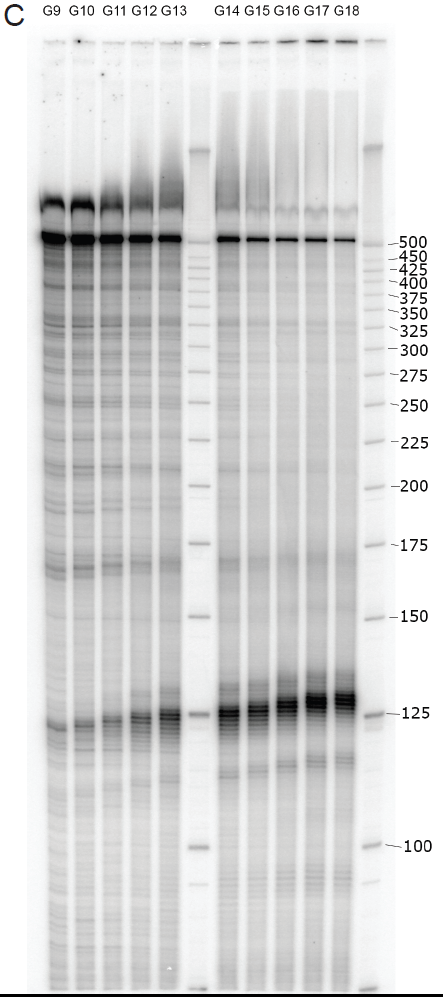


**Supplementary Figure S9: Complete sequencing gels from Figure 5.**

Panels **A-C** correspond to panels in **Figure 5A-C.**


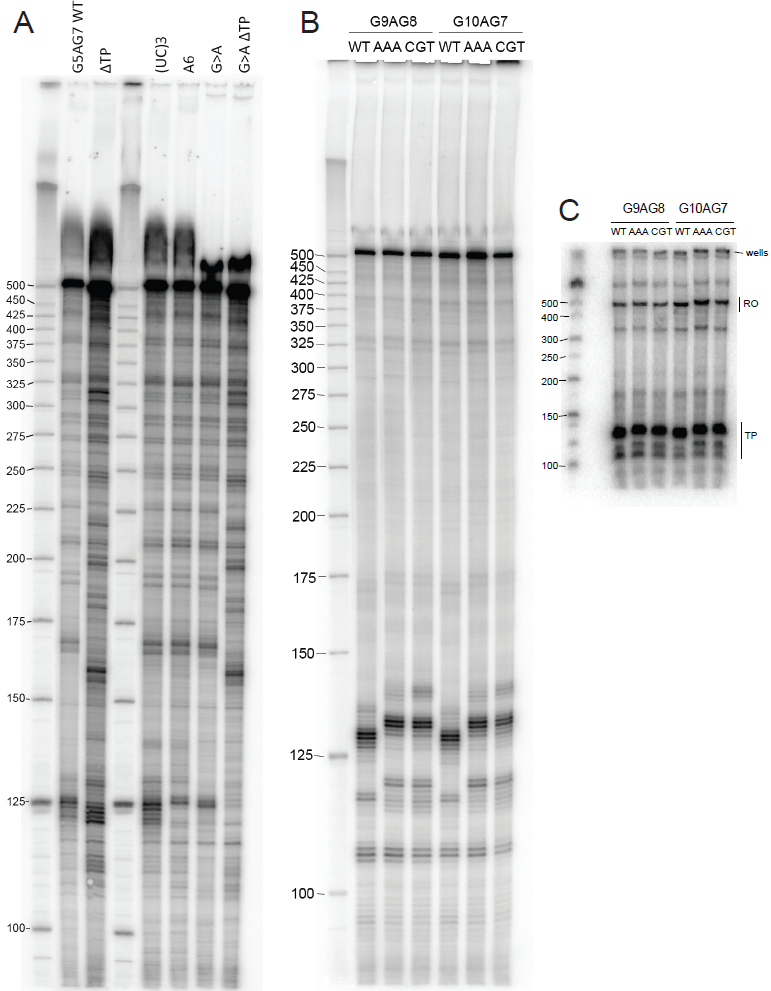


**Supplementary Figure S10: Complete sequencing and mini-gels from Figure 7.**

**A.** Sequencing gel corresponding to scanned data in Figure 6B. **B.** Sequencing gel corresponding to **Figure 6C**. **C.** Representative mini-gel from *in vitro* transcription assay on the substrates in **Figure 6C**.

**Supplementary Figure S11: Complete sequencing gels showing some of the variants in Supplementary Figure S7D not presented elsewhere.**
